# The Fitting Optimization Path Analysis on Scale Missing Data: Based on the 507 Patients of Poststroke Depression Measured by SDS

**DOI:** 10.1155/2022/5630748

**Published:** 2022-01-13

**Authors:** Xiaoying Lv, Ruonan Zhao, Tongsheng Su, Liyun He, Rui Song, Qizhen Wang, Xueyun Yu, Yanbo Zhu

**Affiliations:** ^1^School of Chinese Medicine, Beijing University of Chinese Medicine, Beijing, China; ^2^Institute of Basic Research In Clinical Medicine, China Academy Of Chinese Medical Sciences, Beijing, China; ^3^Xi'an Encephalopathy Hospital of Traditional Chinese Medicine, Shanxi University of Chinese Medicine, Xi'an, China; ^4^Shanxi Provincial Hospital of Chinese Medicine, Xi'an, Shanxi, China; ^5^School of Management, Beijing University of Chinese Medicine, Beijing, China

## Abstract

**Objective:**

To explore the optimal fitting path of missing data of the Scale to make the fitting data close to the real situation of patients' data.

**Methods:**

Based on the complete data set of the SDS of 507 patients with stroke, the data simulation sets of Missing Completely at Random (MCAR), Missing at Random (MAR), and Missing Not at Random (MNAR) were constructed by R software, respectively, with missing rates of 5%, 10%, 15%, 20%, 25%, 30%, 35%, and 40% under three missing mechanisms. Mean substitution (MS), random forest regression (RFR), and predictive mean matching (PMM) were used to fit the data. Root mean square error (RMSE), the width of 95% confidence intervals (95% CI), and Spearman correlation coefficient (SCC) were used to evaluate the fitting effect and determine the optimal fitting path.

**Results:**

when dealing with the problem of missing data in scales, the optimal fitting path is ① under the MCAR deletion mechanism, when the deletion proportion is less than 20%, the MS method is the most convenient; when the missing ratio is greater than 20%, RFR algorithm is the best fitting method. ② Under the Mar mechanism, when the deletion ratio is less than 35%, the MS method is the most convenient. When the deletion ratio is greater than 35%, RFR has a better correlation. ③ Under the mechanism of MNAR, RFR is the best data fitting method, especially when the missing proportion is greater than 30%. In reality, when the deletion ratio is small, the complete case deletion method is the most commonly used, but the RFR algorithm can greatly expand the application scope of samples and save the cost of clinical research when the deletion ratio is less than 30%. The best way to deal with data missing should be based on the missing mechanism and proportion of actual data, and choose the best method between the statistical analysis ability of the research team, the effectiveness of the method, and the understanding of readers.

## 1. Introduction

The scale data is filled in by doctors or patients according to the situation and patient feelings at that time. Once it is missing, it is difficult to trace the original case and verify the data [1–3]. However, due to various reasons, such as the privacy and specialty of the scale items, scale data missing often occurs in the process of medical research. For example, some scales involve privacy items, and some patients adopt an avoidance attitude when facing sensitive problems. Some items of the scale may involve traditional Chinese medicine, clinical or other medical terms. Sometimes the patients did not answer because they did not understand the meaning of the question or option—missing data due to the negligence of respondents [4]. However, in previous studies, few researchers have discussed the filling method suitable for scale data missing, and there is no final conclusion on which method is more suitable for fitting scale data. This problem is particularly prominent in the data of medical, psychological measurement scales. For example, a large prospective cohort study of 71412 women was conducted in France. Data including the center for epidemiological studies depression (CES-D) were collected, of which 45% had missing entries in the scale [5]. A program conducted a questionnaire survey on mental health among 2919 sixth graders in 21 schools across the country, and 86% of the students missed one or more variables [6].

Poststroke depression (PSD) refers to a mood disorder characterized by continuous depression and decreased interest after stroke. It is one of the common complications of a stroke. It is mainly manifested in a series of mental disorders such as depression and slow thinking and even seriously affects the quality of life and rehabilitation of such patients. Zung's Self-rating Depression Scale (SDS) is a 4-level self-rating scale with 20 items, which can directly reflect the subjective feelings of depressed patients and the changes in their depression status in the process of diagnosis and treatment [7]. Therefore, 507 cases of poststroke depression data were selected for fitting study to analyze the optimal path of missing data of the scale.

Data missing mechanisms describe the possible relationship between missing values and observed variables, which can be divided into the following three types: Missing Completely at Random (MCAR), Missing at Random (MAR), and Missing Not at Random (MNAR). Different data fitting methods should be selected according to different missing mechanisms [8]. The contemporary data missing processing methods can make more in-depth and effective use of collected clinical data information to carry out multiple interpolation fitting of missing data, so as to make the data more truly reflect the actual clinical situation. However, in the practical application, the researchers have the problem of blindly applying statistical methods to the treatment, failing to consider the characteristics of the experiment and the possible missing mechanism to make an appropriate analytic strategy in advance in the scheme [9]. Meanwhile, different fitting methods will also draw different conclusions under different missing rates [10]. For example, Van Hulse and Khoshgoftaar believe that when the missing rate reaches more than 40%, the utilization value of data is almost lost [11]. Therefore, based on the data of SDS, this study constructed simulated data sets with different missing mechanisms and different missing rates (5%, 10%, 15%, 20%, 25%, 30%, 35%, and 40%), and compared three interpolation methods: Mean Substitution (MS), Random Forest Regression (RFR), and Predictive Mean Matching (PMM), in the aspect of fitting effects on missing data in the SDS scale, to explore the best fitting optimization path for the missing scale data in clinical research, to make the fitting data close to the real situation of the treatment of patients. Also, to improve the efficiency of statistical tests more accurately and provide methodological support for the fitting of missing data in the scale in the future.

## 2. Materials and Methods

### 2.1. Data Information

The study data were derived from complete SDS scale data of 507 stroke subjects collected from more than 10 hospitals in Shandong, Nanjing, and Gansu, from September 2015 to March 2017, including 313 males (61.7%) with an average age of 60.11 ± 0.54, and 194 females (38.3%) with an average age of 62.94 ± 0.63. The study took the above complete data as the training set, and the data set after fitting was called the simulation set.

### 2.2. Simulation Methods

Based on the training set, the NaControl function in R software was applied to construct the simulation data set. The details are as follows:

① Missing Completely at Random (MCAR): the probability of data missing has no relationship with both observed data and unobserved data [12]. In this study, data were randomly deleted from the training set at the proportions of 5%, 10%, 15%, 20%, 25%, 30%, 35%, and 40%.
② Missing at Random (MAR): the probability of data missing is related to the observed variables but independent of the characteristics of the unobserved data [13]. One study showed a significant increase in the number of missing items on the SDS depression scale in male subjects [14]. In order to facilitate the comparison of the three data missing methods under the eight missing rates, the data of 194 female subjects were randomly deleted at a proportion of 10%, and the data of the remaining 313 male patients were randomly deleted at a certain proportion. The datasets of male and female subjects were transformed into simulated sets with missing ratios of 5%, 10%, 15%, 20%, 25%, 30%, 35%, and 40%, see Table 1.
③ Missing Not at Random (MNAR): the probability of data missing is related to the observed data itself [15]. Zung, the author of the SDS, believed that among depressed patients, the severity of the perception of diurnal change (item 2) was most closely related to the degree of depression [7]. The patients with a score of 3 or 4 in item 2 of the SDS had more severe depression. This study considered the possibility that the more depressed the subjects were, the more likely their data would be missing. Therefore, the data of 108 subjects scoring 1 or 2 points in item 2 were randomly deleted at a proportion of 10%, and the data of 399 subjects scoring 3 or 4 points were constructed according to the proportion of missing data. The two were combined into a simulation set with the above-mentioned missing ratio. See Table 1.

In conclusion, a simulated missing data set was constructed for each training set, and three fitting methods were applied to fitting the data set. Three missing mechanisms (MCAR, MAR, and MNAR, respectively, expressed in C, A, N), eight missing ratios (5%, 10%, 15%, 20%, 25%, 30%, 35%, and 40%, respectively, in 5, 10, 15, 20, 25, 30, 35, and 40), and three interpolation methods (MS, PMM, and RFR, respectively, in M, P, and R) were used to deal with the data sets. For example, the fitting set C10M is a data set that is formed after the simulation set with a missing ratio of 10% is fitted by Mean Substitution under the MCAR mechanism.

### 2.3. Missing Value Processing Methods and Some Principles

#### 2.3.1. Mean Substitution

The Mean Substitution refers to that the mean value of nonmissing data of key variables is used as the substitute value of missing data. In the process of filling in the missing values, only one substitute value for missing values is generated. That is, the mean value of the observed data in this sample is used as the substitute value so as to generate a complete data set for analysis [16, 17]. This method applies the HMISC function of R software, and its parameters are set as follows: impute (data, fun = mean).

#### 2.3.2. Predictive Mean Matching

The PMM method is one of the methods to fill in the data of monotone missing continuous variables. This method assumes that there is a linear regression relationship between missing data and nonmissing data. By establishing the regression model of both sides and randomly selecting m parameters from the posterior distribution of regression coefficient estimates, the predicted values are obtained through the calculations of these parameters and used to replace the missing values [18, 19]. This method is suitable for continuous variables in monotone deletion mode. The mice function of R software is used in this method, and the parameters are set as follows: mice (data, *m* = 20, maxit = 5, meth = “pmm,” seed = 500).

#### 2.3.3. Random Forest Regression

Random Forest algorithm is an integrated learning algorithm proposed by Breiman in 2001, which is used to solve the problems of classification prediction, regression prediction, and feature selection of high-dimensional nonlinear data. RMR method is an improved algorithm of bagging algorithm. It uses K classification and regression decision trees (CART) as the base learner [20] and takes the average of the predicted values of *K* base learners as the final result [21, 22]. When dealing with missing data, it is less affected by outliers and has no restrictions on the distribution of data. It can effectively analyze high-dimensional complex data [23]. The missForest function of R software is used in this method, and the parameters are set as follows: missForest (data, ntree = 50).

### 2.4. Evaluation Methods and Indicators

The evaluation indexes were the root mean square error (RMSE), the width of 95% confidence intervals (95% CI), and Spearman correlation coefficient (SCC) obtained by paired T-test of the total scores of the training set and the simulation set [24]. The smaller the value range of RMSE, the better the fitting accuracy [25]. The narrower the 95% CI, the higher the fitting precision [26]. The bigger the value of the Spearman correlation coefficient, the better correlation of the fitting data [27].

## 3. The Results

### 3.1. Number of Missing Items

With the increase of the proportion of data missing in the SDS, the number of missing items and samples gradually increased. When the missing ratio was less than 10%, the number of missing items in most subjects' SDS was concentrated in 1 to 3. When the missing ratio was from 10% to 20%, the result in most subjects' SDS concentrated in 3 to 5. When the data missing ratio reached 40%, the number of missing items in most subjects could be controlled below 10 under the MCAR mechanism, while in the MAR mechanism, 50% subjects' numbers were greater than 10. See Tables 2–4 for more details.

### 3.2. Comparisons of Fitting Effects

#### 3.2.1. Root Mean Square Error

**Figure 1:**
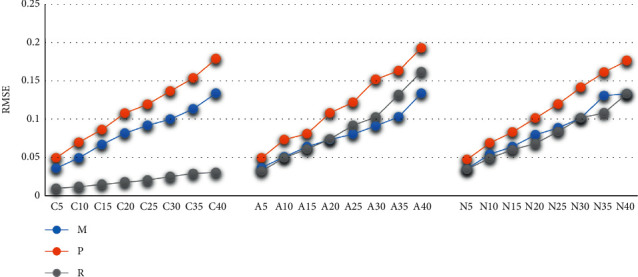
The evaluations of fitting effects by RMSE.

The RMSE is an indicator of fitting accuracy. The fitting results of the three methods show that, with the increase of the missing ratio, the fitting accuracy becomes lower and lower, and the value of RMSE is between 0 and 0.2. Compared with MS and PMM, the RMSE value of RFR is the lowest under the three deletion mechanisms, especially under the MCAR mechanism, the RMSE is always less than 0.1. When the deletion ratio is greater than 30%, the RMSE of the three methods is greater than 0.1. When the deletion ratio is less than 20%, the RMSE value of RFR is the lowest; under the MNAR mechanism, when the deletion ratio is greater than 30%, the result of RFR is also better. The accuracy of PMM is lower than the other two methods. See Figure 1.

#### 3.2.2. The Width of 95% Confidence Intervals

**Figure 2:**
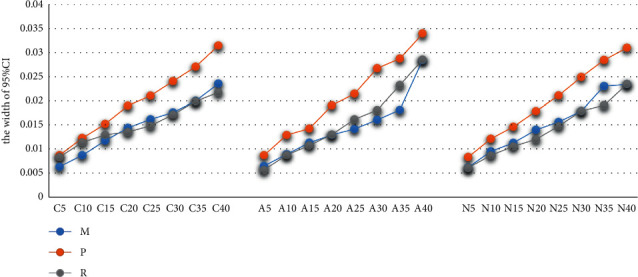
The evaluations of fitting effects by the width of 95% CI.

Usually, the 95% confidence interval stands for the fitting precision. With the increase of deletion ratio, 95% CI width becomes higher and higher, and the value of 95% CI width is between 0.005 and 0.035. Under the MCAR mechanism, when the deletion ratio is less than 15%, the width of 95% CI of MS is the narrowest, and the width 95% CI of MS under the MAR mechanism is also narrower than the other two methods. However, under the MNAR mechanism, the fitting precision of RFR is better than the other two methods. When the deletion ratio is less than 20%, the width of 95% CI of MS and the width of 95% CI of RFR are less than 0.015. For more details; see Figure 2.

#### 3.2.3. Spearman Correlation Coefficient

**Figure 3:**
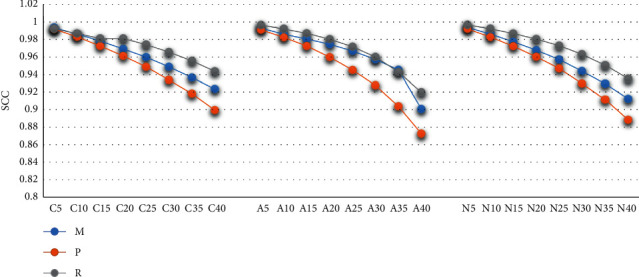
The evaluations of fitting effects by SCC.

SCC is an indicator representing the fitting correlation. With the increase of deletion ratio, the correlation becomes lower and lower, and the value of SCC is between 1 and 0.85. Under the MCAR mechanism, although the missing ratio reaches 40%, the SCC of the fitting set and the training set can still reach 0.9. When the deletion ratio is less than 20%, the SCC value of the three methods can reach 0.95. When the deletion ratio is less than 30%, the SCC of RFR can still be greater than 0.95, and the degree of correlation is higher. See Figure 3 for details.

## 4. Discussion

In this paper, three missing mechanisms, eight missing ratios, and three missing value processing methods (MS, PMM, and RFR) were set to fill the data gaps in the SDS. The SDS has 20 items of self-rating questions and the correlations between items are good, which is easier to lose data due to various reasons such as incomprehension of questions, avoidance of sensitive questions, loss of follow-up, and so on [28]. In addition, the absence of data will cause the reduction of effective data, increase the confidence interval, affect the statistical analysis, and eventually lead to the bias of study results, which may draw conclusions that are not consistent with the facts [4]. Therefore, an effective way to deal with the problem of data missing is in need.

In clinical research, too many missing items on the scale may result in inaccurate or biased estimations. For example, some researchers believed that the validity of statistical analysis would be significantly reduced when one-third of the items in the SF-12 scale was missing [28]. In the study on data missing of the Unified Dyskinesia Rating Scale (UDysRS), some other researchers found that if the number of missing items reached 8 or more, the validity of statistical analysis towards study results would be lost [29], indicating that the missing items of the scale should be controlled within a certain range so as not to affect the final statistical results. In this study, when the missing ratio of SDS was small (5%, 10%), the number of missing items in the SDS for most subjects was below or equal to 5. When the missing ratio reached 30%, the numbers in most patients concentrated in 5 to 8. While, as the proportion increased to 35% or 40%, a small number of subjects even had missing items of 18. Therefore, in the actual research, if the data missing ratio is greater than 30%, attention should be paid to the subjects' responses to the scale items. If there are subjects with many missing items in the scale, researchers should consider removing them from the study to ensure the reliability of the evaluation results as much as possible.

It can be seen from this study that in most simulation scenarios, the fitting result of the RFR algorithm is closest to the training set and has the best fitting effect in terms of fitting accuracy, precision, and correlation, especially under the mechanism of MCAR. The RFR algorithm does not need to consider the distribution of variables. It is a nonparametric estimation algorithm, which is more widely used [30, 31]. Some researchers believe that the fitting effect of the missing forest is worse than other methods, such as the MS method, k nearest distance filling, or Multivariable Interpolation by chained equations (mice) [32, 33].

Furthermore, when the deletion ratio is less than 20%, the RMSE results of MCAR are given, and the results of other RFRs are very close to those of Ms.; however, when the deletion ratio is larger, RFR reflects better fitting accuracy and correlation. Therefore, the RFR algorithm is the most suitable fitting method for the SDS scale in this study. At the same time, it is also found that with the increase of the missing ratio, the accuracy, precision, and correlation of the fitting set decrease. When the missing ratio is less than 30%, RFR or MS can also control results to a relatively good extent (RMSE <1.0, 95% CI width <0.02, SCC >0.95). Therefore, in practical research, we should pay attention to the proportion and mechanism of data loss and select appropriate data fitting methods.

Among the three missing mechanisms, the MCAR, under which the fitting effect of the three interpolation methods was better than that of the other cases, and the smaller the missing ratio, the closer the fitting result was to the actual value, was the optimal one. The case of the MAR was more complex for the simple reason that it is kind of missing was directly related to the missing of other variables and the data simply could not be processed by the straightforward elimination or the method of Single Imputation. Otherwise, it would cause the bias of study results [4]. The RFR algorithm was more suitable for this case. When it comes to the most complicated MNAR mechanism, the results in this paper showed that the fitting effect of the three interpolation methods, in this case, was not as good as that of under the other two mechanisms, but the RFR algorithm also had some good performances under MNAR.

This study had some limitations. The range of the missing ratio of simulation set in this study is 5%∼40%, and a specific ratio of simulation data missing is selected at an interval of 5%. According to previous studies, it is found that the proportion of missing data is less than 5%, and more accurate analysis results can be obtained by using the deletion method, while more than 40% of missing data fitting will not get more available results, and more literature use inconsistent fitting methods between 5% and 40%. It is difficult for researchers to choose which fitting method to use, and the results are more accurate [34–37]. In the practical application of the SDS scale, the missing ratio should be regarded as a parameter to determine the missing fitting path. Due to the ideal state of missing ratios and missing mechanisms, the simulated dataset in this paper might be slightly different from that in the actual situation, so the study results might not reflect the data missing patterns in real studies as well as possible. In addition, this study only focused on the SDS and the data missing fitting path is only based on the simulation inference of poststroke depression data, which has not been applied to the actual clinical process. The fitting effect needs to be further verified. In addition, in this study, the fitting results of PMM do not show any advantages over the fitting results of MS and RFR, which may be related to the number of iterations set by PMM. In this study, the number of iterations is set to 5. The results may be better if it is set to 50 or more, but this method costs more than the running time of R software of MS and RFR and is more complex. Therefore, from the perspective of time cost, other methods can be preferred. At last, PMM is suitable for continuous variables in monotone deletion mode. This may also be a reason why the PMM results in this article are not very good.

## 5. Conclusions

To sum up, in this study, when dealing with the problem of missing data in scales, the optimal fitting path is ① under the MCAR deletion mechanism, when the deletion proportion is less than 20%, the MS method is the most convenient; when the missing ratio is greater than 20%, the RFR algorithm is the best fitting method. ② Under the Mar mechanism, when the deletion ratio is less than 35%, the MS method is the most convenient. When the deletion ratio is greater than 35%, RFR has a better correlation. ③ Under the mechanism of MNAR, RFR is the best data fitting method, especially when the missing proportion is greater than 30%. In reality, when the deletion ratio is small, the complete case deletion method is the most commonly used, but the RFR algorithm can greatly expand the application scope of samples and save the cost of clinical research when the deletion ratio is less than 30%. The best way to deal with data missing should be based on the missing mechanism and proportion of actual data, and choose the best method between the statistical analysis ability of the research team, the effectiveness of the method, and the understanding of readers.

## Figures and Tables

**Table 1:** Missing ratios of the simulation sets.

Mechanism		5 (%)	10%	15 (%)	20 (%)	25 (%)	30 (%)	35 (%)	40 (%)
MCAR (*n* = 507)		5	10%	15	20	25	30	35	40
MAR	Female (*n* = 194)	10	10	10	10	10	10	10	10
	Male (*n* = 313)	1.90	10.00	18.10	26.20	34.30	42.40	50.50	58.59

MNAR	1-2 points (*n* = 108)	10	10	10	10	10	10	10	10
	3-4 points (*n* = 399)	3.65	10.00	16.35	22.71	29.06	35.41	41.77	48.12

**Table 2:** The item missing conditions of the simulation sets under MCAR (*N* = 507).

Number of missing items (*n* (%))	5%	10%	15%	20%	25%	30%	35%	40%
0	187 (36.9)^a^	58 (11.4)	17 (3.4)	7 (1.4)	1 (0.2)	1 (0.2)	0 (0)	0 (0)
1	183 (73.0)	146 (40.2)^a^	82 (19.6)	33 (7.9)	12 (2.6)	6 (1.4)	0 (0)	1 (0.2)
2	94 (91.5)^b^	135 (66.8)	101 (39.5)^a^	66 (20.9)	34 (9.3)	12 (3.7)	3 (0.6)	2 (0.6)
3	36 (98.6)	96 (85.7)^b^	134 (65.9)	114 (43.4)^a^	79 (24.9)	38 (11.2)	18 (4.1)	7 (2.0)
4	7 (100.0)	49 (95.4)	85 (81.7)^b^	102 (63.5)	80 (40.6)^a^	59 (22.9)	36 (11.2)	21 (6.1)
5	0 (0)	17 (98.8)	53 (92.2)	92 (81.7)^b^	104 (61.1)	107 (44.0)^a^	60 (23.1)	41 (14.2)
6	0 (0)	5 (99.8)	21 (96.3)	54 (92.3)	88 (78.5)^b^	79 (59.6)	93 (41.4)^a^	57 (25.4)^a^
7	0 (0)	1 (100.0)	9 (98.1)	22 (96.6)	56 (89.5)	92 (77.7)^b^	91 (59.4)	74 (40.0)
8	0 (0)	0 (0)	4 (98.9)	12 (99.0)	29 (95.3)	46 (86.8)	93 (77.7)^b^	83 (56.4)
9	0 (0)	0 (0)	1 (100.0)	4 (99.8)	16 (98.4)	43 (95.3)	56 (88.8)	96 (75.3)^b^
10	0 (0)	0 (0)	0 (0)	0 (0)	6 (99.6)	13 (97.8)	35 (95.7)	60 (87.1)
11	0 (0)	0 (0)	0 (0)	1 (100.0)	2 (100.0)	9 (99.6)	13 (98.2)	42 (95.4)
12	0 (0)	0 (0)	0 (0)	0 (0)	0 (0)	1 (99.8)	8 (99.8)	12 (97.8)
13	0 (0)	0 (0)	0 (0)	0 (0)	0 (0)	1 (100.0)	1 (100.0)	7 (99.2)
14	0 (0)	0 (0)	0 (0)	0 (0)	0 (0)	0 (0)	0 (0)	4 (100.0)

^a^The number of missing samples accounts for 25% of the total. ^b^The number of missing samples accounts for 75% of the total.

**Table 3:** The item missing conditions of the simulation sets under MAR (*N* = 507).

Number of missing items (*n* (%))	5%	10%	15%	20%	25%	30%	35%	40%
0	225 (44.4)^a^	58 (11.4)	26 (5.1)	18 (3.6)	18 (3.6)	18 (3.6)	18 (3.6)	18 (3.6)
1	146 (73.2)	129 (36.9)^a^	82 (21.3)	64 (16.2)	57 (14.8)	56 (14.6)	56 (14.6)	56 (14.6)
2	71 (87.2)^b^	166 (69.6)	113 (43.6)^a^	76 (31.2)^a^	63 (27.2)^a^	62 (26.8)^a^	58 (26.0)^a^	58 (26.0)^a^
3	46 (96.3)	95 (88.4)^b^	110 (65.3)	81 (47.1)	53 (37.7)	46 (35.9)	43 (34.5)	43 (34.5)
4	12 (98.6)	37 (95.7)	70 (79.1)^b^	69 (60.7)	40 (45.6)	20 (39.8)	13 (37.1)	12 (36.9)
5	4 (99.4)	15 (98.6)	58 (90.5)	66 (73.8)	51 (55.6)	20 (43.8)	8 (38.7)	6 (38.1)
6	2 (99.8)	5 (99.6)	31 (96.6)	58 (85.2)^b^	43 (64.1)	25 (48.7)	15 (41.6)	5 (39.1)
7	1 (100.0)	1 (99.8)	16 (99.8)	33 (91.7)	63 (76.5)^b^	49 (58.4)	19 (45.4)	4 (39.8)
8	0 (0)	1 (100.0)	1 (100.0)	21 (95.9)	58 (88.0)	62 (70.6)	26 (50.5)	10 (41.8)
9	0 (0)	0 (0)	0 (0)	10 (97.8)	34 (94.7)	54 (81.3)^b^	57 (61.7)	27 (47.1)
10	0 (0)	0 (0)	0 (0)	8 (99.4)	14 (97.4)	41 (89.3)	56 (72.8)	45 (56.0)
11	0 (0)	0 (0)	0 (0)	3 (100.0)	9 (99.2)	23 (93.9)	50 (82.6)^b^	45 (64.9)
12	0 (0)	0 (0)	0 (0)	0 (0)	2 (99.6)	24 (98.6)	44 (91.3)	57 (76.1)^b^
13	0 (0)	0 (0)	0 (0)	0 (0)	1 (99.8)	5 (99.6)	24 (96.1)	57 (87.4)
14	0 (0)	0 (0)	0 (0)	0 (0)	1 (100.0)	2 (100.0)	13 (98.6)	30 (93.3)
15	0 (0)	0 (0)	0 (0)	0 (0)	0 (0)	0 (0)	4 (99.4)	17 (96.6)
16	0 (0)	0 (0)	0 (0)	0 (0)	0 (0)	0 (0)	1 (99.6)	13 (99.2)
17	0 (0)	0 (0)	0 (0)	0 (0)	0 (0)	0 (0)	1 (99.8)	4 (100.0)
18	0 (0)	0 (0)	0 (0)	0 (0)	0 (0)	0 (0)	1 (100.0)	0 (0)

^a^The number of missing samples accounts for 25% of the total. ^b^The number of missing samples accounts for 75% of the total.

**Table 4:** The item missing conditions of the simulation sets under MNAR (*N* = 507).

Number of missing items (*n* (%))	5%	10%	15%	20%	25%	30%	35%	40%
0	171 (33.7)a	61 (12.0)	23 (4.5)	15 (3.0)	15 (3.0)	13 (2.6)	13 (2.6)	13 (2.6)
1	177 (68.6)	146 (40.8)a	76 (19.5)	40 (10.8)	33 (9.5)	31 (8.7)	30 (8.5)	30 (8.5)
2	98 (88.0)b	143 (69.0)	115 (42.2)a	69 (24.5)	40 (17.4)	33 (15.2)	32 (14.8)	32 (14.8)
3	43 (96.4)	91 (87.0)b	112 (64.3)	75 (39.3)a	53 (27.8)a	29 (20.9)	23 (19.3)	19 (18.5)
4	12 (98.8)	43 (95.5)	83 (80.7)b	98 (58.6)	63 (40.2)	36 (28.0)a	15 (22.3)	13 (21.1)
5	2 (99.2)	16 (98.6)	52 (90.9)	84 (75.1)b	83 (56.6)	59 (39.6)	16 (25.4)a	6 (22.3)
6	4 (100.0)	7 (100.0)	32 (97.2)	61 (87.2)	76 (71.6)	82 (55.8)	48 (34.9)	27 (27.6)a
7	0 (0)	0 (0)	11 (99.4)	32 (93.5)	64 (84.2)b	72 (70.0)	59 (46.5)	31 (33.7)
8	0 (0)	0 (0)	2 (99.8)	18 (97.0)	43 (92.7)	57 (81.3)b	78 (61.9)	67 (46.9)
9	0 (0)	0 (0)	1 (100.0)	9 (98.8)	24 (97.4)	43 (89.7)	85 (78.7)b	54 (57.6)
10	0 (0)	0 (0)	0 (0)	3 (99.4)	9 (99.2)	28 (95.3)	43 (87.2)	81 (73.6)
11	0 (0)	0 (0)	0 (0)	2 (99.8)	4 (100.0)	19 (99.0)	37 (94.5)	55 (84.4)b
12	0 (0)	0 (0)	0 (0)	1 (100.0)	0 (0)	2 (99.4)	16 (97.6)	34 (91.1)
13	0 (0)	0 (0)	0 (0)	0 (0)	0 (0)	2 (99.8)	7 (99.0)	25 (96.1)
14	0 (0)	0 (0)	0 (0)	0 (0)	0 (0)	1 (100.0)	4 (99.8)	16 (99.2)
15	0 (0)	0 (0)	0 (0)	0 (0)	0 (0)	0 (0)	1 (100.0)	1 (99.4)
16	0 (0)	0 (0)	0 (0)	0 (0)	0 (0)	0 (0)	0 (0)	2 (99.8)
17	0 (0)	0 (0)	0 (0)	0 (0)	0 (0)	0 (0)	0 (0)	1 (100.0)

^a^The number of missing samples accounts for 25% of the total. ^b^The number of missing samples accounts for 75% of the total.

## Data Availability

The datasets used during the study are available from the corresponding author on reasonable request.
